# Toward an attentional turn in research on risky choice

**DOI:** 10.3389/fpsyg.2022.953008

**Published:** 2022-09-06

**Authors:** Veronika Zilker, Thorsten Pachur

**Affiliations:** ^1^Center for Adaptive Rationality, Max Planck Institute for Human Development, Berlin, Germany; ^2^TUM School of Management, Technical University of Munich, Munich, Germany

**Keywords:** decision making under risk, risky choice, theory development, attention, cumulative prospect theory (CPT)

## Abstract

For a long time, the dominant approach to studying decision making under risk has been to use psychoeconomic functions to account for how behavior deviates from the normative prescriptions of expected value maximization. While this neo-Bernoullian tradition has advanced the field in various ways—such as identifying seminal phenomena of risky choice (e.g., Allais paradox, fourfold pattern)—it contains a major shortcoming: Psychoeconomic curves are mute with regard to the cognitive mechanisms underlying risky choice. This neglect of the mechanisms both limits the explanatory value of neo-Bernoullian models and fails to provide guidance for designing effective interventions to improve decision making. Here we showcase a recent “attentional turn” in research on risk choice that elaborates how deviations from normative prescriptions can result from imbalances in attention allocation (rather than distortions in the representation or processing of probability and outcome information) and that thus promises to overcome the challenges of the neo-Bernoullian tradition. We argue that a comprehensive understanding of preference formation in risky choice must provide an account on a mechanistic level, and we delineate directions in which existing theories that rely on attentional processes may be extended to achieve this objective.

## 1. Introduction

One of the longest standing puzzles in the decision sciences is how people evaluate and choose between risky options, whose consequences cannot be predicted with certainty. Researchers have tried to address this question primarily by identifying how people deviate from the predictions of a normative economic model, then modifying the model to make it descriptively more valid. For instance, in the St. Petersburg paradox, most people are willing to pay only a moderate amount of money to play a coin toss game that involves risk but has an infinite expected value. Yet expected value (EV) theory would predict that people should be willing to pay a large sum to play the game. To account for this violation of EV theory, expected utility (EU) theory modified EV theory by introducing a concave utility function that assumes diminishing returns by transforming the objective outcomes of options into subjective utilities before weighting them by their probabilities (von Neumann and Morgenstern, 1944; Bernoulli, 1954).

However, human behavior also violates EU theory. For instance, EU theory cannot accommodate the fourfold pattern of risk attitudes (Tversky and Fox, 1995)—the phenomenon that people are risk averse (risk seeking) for high probability gains (losses) and low probability losses (gains). Consequently, prospect theory (PT; Kahneman and Tversky, 1979) and its extension, cumulative prospect theory (CPT; Tversky and Kahneman, 1992), were introduced. CPT modifies EU theory such that the subjective values of outcomes are no longer assumed to be weighted by their objective probabilities; instead, an inverse S-shaped probability weighting function overweights low-probability events and underweights medium- to high-probability events. The shape of the probability weighting function is characterized by its curvature (indicating the decision maker's probability sensitivity) and its elevation (indicating the decision maker's optimism/pessimism). Moreover, probability weighting in CPT depends on an event's rank among all possible events. In CPT, EU theory's utility function is replaced by a value function with a reference point; the function is concave (convex) for gains (losses) and steeper in the loss than in the gain domain, implementing the assumption of loss aversion. We refer to models such as EU theory and CPT—which use psychoeconomic functions (i.e., nonlinear utility and probability weighting functions) to account for violations of EV maximization—as *neo-Bernoullian* models. The approach of identifying behavioral deviations from EV and EU theory and capturing them in psychoeconomic functions has been highly influential, both in behavioral economics and in psychology, and has helped identify and characterize several key regularities of risky choice (see also Birnbaum, 2008).

The neo-Bernoullian modeling tradition, however, has an important downside: Its main concern is to account for choice behavior, with only little interest in the underlying psychological processes (Friedman and Savage, 1948). This imposes limitations. For instance, without understanding the cognitive processes underlying people's decisions it is impossible to design effective interventions to mitigate deviations from benchmarks such as EU or EV maximization (Weber and Johnson, 2009; Payne and Venkatraman, 2010). To illustrate, someone who ignores probabilities and someone who fails to understand probabilities would deviate from choices predicted based on an objective treatment of probabilities in similar ways but require a different intervention to rectify these deviations. Moreover, without a theoretical account of the cognitive processes it is difficult to specify at which stage and how psychological variables (e.g., affect; Lerner and Keltner, 2001; Pachur et al., 2014; Suter et al., 2016) or features of the choice context (e.g., whether people learn about the options via description or experience; Hertwig and Erev, 2009), can modulate risky choice behavior. For instance, characterizing patterns in description-based and experience-based choice with psychoeconomic functions indicates notable differences in probability weighting between these two learning modes (Wulff et al., 2018). Understanding the mechanisms responsible for this gap in choice is difficult without a model that also explains the underlying information processing.

In this article we review recent progress toward overcoming the neglect of mechanisms that characterizes neo-Bernoullian models. Decades ago, Simon (1978) highlighted the key role attention plays in understanding decision making, but only recently have attempts to integrate attentional mechanisms in computational process models of decision making started to emerge. Here we review work showcasing that attentional processes can provide relatively simple yet powerful mechanistic explanations for longstanding puzzles in research on risky choice, and we delineate how these process-level insights can be linked to characteristic distortions in psychoeconomic functions. We discuss remaining questions and argue that attentional processes should be integrated more comprehensively in theories of risky choice. We begin by outlining how research on riskless choice has started to uncover how attentional processes modulate preference formation.

## 2. Preference construction and attention

In contrast to research on risky choice, a more process-oriented approach has been more readily adopted in research on riskless choice, such as choosing among food items (Shimojo et al., 2003; Krajbich et al., 2010). Measures of information search (e.g., eye tracking) have revealed some striking regularities—for instance, people tend to increasingly look at the item they ultimately choose over the time course of choice (the gaze cascade; Shimojo et al., 2003), and people are more likely to choose an item if they look at it longer than at the alternative (e.g., Krajbich et al., 2010; Zilker, 2022). These phenomena suggest a tight coupling between attention and preference formation.

Cognitive processes that might lead to these phenomena were formalized in computational models, which—unlike neo-Bernoullian theories—operate on the level of cognitive processing. An influential model of this type, the attentional drift diffusion model (aDDM; Krajbich et al., 2010), posits the decision process as an accumulation of evidence on the options over time and assumes that the accumulation of evidence toward an option is amplified (relative to the unattended option) whenever this option is attended to. The aDDM accounts for the gaze cascade and the attention–preference link described above. Investigations spanning various domains of decision making—including choices between monetary and food items, and even social decision making—have revealed that this attentional mechanism also seems to be at work in risky choice (Smith and Krajbich, 2018). Nevertheless, research on the aDDM has not addressed how attentional processes in risky choice might relate to the systematic deviations from EV and EU maximization carved out by the neo-Bernoullian tradition. Recent work has started to bridge this gap between research traditions (thereby also contributing to theory integration; e.g., Gigerenzer, 2017; Pachur, 2020).

## 3. Linking psychoeconomic constructs in risky choice to imbalances in attention

Neo-Bernoullian models rely on psychoeconomic functions (e.g., nonlinear value and weighting functions) to account for deviations from EV and EU maximization. Recent work has uncovered that variability in predecisional attention allocation—measured, e.g., using eye tracking—may explain how the characteristic shapes of these functions come about. Using the process-tracing tool Mouselab (Payne et al., 1993), Pachur et al. (2018) measured how long participants inspected information on the attributes of risky options before making a choice. They modeled participants' choices with CPT and related the resulting parameter estimates to the attentional measures. The estimated value functions were more strongly curved for participants who inspected outcome information for a shorter time than for participants who inspected outcome information for a longer time. Furthermore, the estimated probability weighting functions were more strongly curved for participants who inspected probability information for a shorter time than for participants who inspected probability information for a longer time. These findings indicate that attention allocation to specific attributes in risky choice may modulate how severely people deviate from EV and EU maximization.

In addition, there are theory-driven analyses of how attentional mechanisms might relate to CPT's constructs. We (Zilker and Pachur, 2021) linked the mechanism proposed in the aDDM to nonlinear probability weighting. Specifically, we used the aDDM to simulate choices for binary choice problems and varied the strength and direction of attentional biases to one of the options in the choice problem. The simulated choices were modeled with CPT. The choice patterns arising from attentional biases in information search were reflected in highly systematic differences in the shape of the estimated probability weighting functions. For instance, when attention was biased to the safe option in a choice between a safe and a risky option, the resulting probability weighting functions were less elevated and more strongly curved—indicating a stronger overweighting of certainty—than when attention was biased to the risky option.

These results point to a process-level, mechanistic explanation for choice patterns that are commonly described with CPT's probability weighting function. Notably, the aDDM gives rise to these distortions in probability weighting merely by assuming attentional biases during evidence accumulation, without applying any nonlinear distortion to the options' outcomes or probabilities. This highlights that choice patterns captured by distorted psychoeconomic functions can arise even when the attributes of choice problems are processed and evaluated in a non-distorted manner.

The analyses presented in Zilker and Pachur (2021) reveal how deviations from maximization might be linked to biases in attention allocation across options. To test whether such a link holds empirically, we reanalyzed a large pool of data from previous process-tracing studies. Indeed, attentional imbalances between options during predecisional information search were associated with specific distortions in probability weighting (Zilker and Pachur, 2021).

Using a similar approach but a different class of cognitive process models, heuristics, Pachur et al. (2017) analyzed how imbalances in attention allocation across attributes in risky choice might be related to the shape of psychoeconomic functions. For instance, some heuristics (e.g., minimax and maximax) focus on outcome information only and ignore probabilities; other heuristics (e.g., the least-likely heuristic) consider both outcome and probability information. By modeling choices predicted by heuristics with CPT, the authors showed that the different attentional policies implied by various heuristics are linked with specific distortions in the shape of CPT's psychoeconomic functions.

The insights obtained by these analyses help alleviate the neglect of mechanisms in neo-Bernoullian theories. They also suggest novel, process-based explanations for the impact of contextual variables (e.g., learning about options via description or experience) on psychoeconomic functions. For instance, people might show systematically different attentional biases depending on whether they learned about the options from description or experience, which might explain the description-experience gap in terms of probability weighting. Likewise, other psychological variables known to modulate risky decision making (e.g., affect) might operate by modulating the attentional process (e.g., Fehr-Duda et al., 2011). Moreover, the analyses point toward novel ways of designing interventions that might render probability weighting more objective (i.e., linear). Specifically, if preferences deviating from linear weighting can be attributed to attentional biases, a greater adherence to objective weighting might be achieved by manipulations (e.g., attentional cues) that lead to a more balanced allocation of attention across attributes and options.

In addition to the attention-based approaches to modeling risky choice originating in cognitive psychology, recent research in behavioral economics has also contributed to elaborating this link. For instance, Smith et al. (2019) propose a random utility approach for estimating the impact of attention on preferences. Similarly, Engelmann et al. (2021) integrate prominent economic theories—salience theory and rational inattention (Sims, 2003; Bordalo et al., 2012)—to disentangle bottom-up and top-down effects of attention on economic decisions. Overall, the quest for a unified theory of the attentional roots of decision making under risk will thus be an interdisciplinary one, involving both cognitive psychology, behavioral economics, and neuroscience. Moreover, it is pertinent to note that attention is not the only cognitive process that can modulate decision making under risk. Memory, executive functions, and learning processes may shape risky decisions as well.

## 4. Discussion

We have reviewed empirical and theoretical work revealing how attentional processes can explain the risky choice phenomena that shaped the development of neo-Bernoullian theories. Although these analyses have enriched the understanding of how patterns in attention allocation relate to the shapes of psychoeconomic functions, existing theoretical accounts of the attentional process in risky decision making are incomplete. Perhaps most importantly, relatively little is known about how attentional biases in risky choice come about in the first place. Ideally, a comprehensive theoretical account on the level of cognitive processing should be able to predict (a) how attention is initially allocated to the different attributes in a given choice problem, (b) how attention allocation and potential biases therein unfold during evidence accumulation, (c) how a preference is formed based on the sampled information about the options' outcomes and probabilities, and how this process is modulated by attention, and ultimately, (d) how a choice is made. We next outline possible starting points for theorizing about attention allocation in risky choice.

### 4.1. Attention guided by accumulated evidence

Although the aDDM is a simple and elegant tool for explaining how imbalances in attention allocation can lead to deviations from EV or EU maximization, it does not predict attention allocation itself. How might imbalances in attention allocation come about? One possibility is that such imbalances emerge dynamically during evidence accumulation. If information search is guided by the amount of evidence accumulated for an option at a given time, attention allocation might reflect differences between the options in the evidence accumulated thus far (e.g., Gluth et al., 2020; Callaway et al., 2021; Glickman et al., 2022). The observation that options sometimes tend to capture attention proportional to their value (Anderson et al., 2011; Le Pelley et al., 2016; Gluth et al., 2018) seems consistent with this possibility. However, unless accompanied by further factors that shape attention allocation, value-based attention may not be able to explain systematic deviations from EV or EU maximization—the more valuable option would almost necessarily end up being attended to more and would thus be more likely to be chosen.

### 4.2. Attention guided by features of the choice problem

Unbalanced attention allocation might also be driven by specific features of the options—leading to regularities such as the Allais paradox and the fourfold pattern. Among such stimulus features might be the size and salience of stimuli (Orquin et al., 2021). Although in standard paradigms font size and display features are usually kept constant (but see Weber and Kirsner, 1997), the options might still differ in visual properties. For instance, when people choose between a safe option and a risky option—where the latter usually consists of more information than the former—the options differ in complexity (Zilker et al., 2020). These differences in complexity may lead to differences in attention allocation (Orquin and Loose, 2013), which in turn might affect evidence accumulation and choice.

Even when the options do not differ in visual complexity, differences in their riskiness might still modulate attention. For instance, it has been proposed that options with higher variance are associated with more extensive internal sampling (Johnson and Busemeyer, 2005). To the extent that external attention also reflects internal sampling processes, this might lead to attentional imbalances between options. Recent formal models posit that people may predominantly direct their attention to the option whose outcome distribution is more variable, thus reducing uncertainty about its value (Callaway et al., 2021; Jang et al., 2021). Consistently, in paradigms in which people learn about the options based on free sampling from the options' outcome distributions, they tend to draw more samples from the option with higher variance (Lejarraga et al., 2012; Pachur and Scheibehenne, 2012).

Further, attention seems to be sensitive to the magnitude of the attribute values. For instance, larger outcomes or probabilities tend to receive more attention than smaller ones (e.g., Fiedler and Glöckner, 2012). According to decision field theory (Busemeyer and Townsend, 1993), an outcome should receive more attention the more likely it is to occur (see also Bhatia, 2014), but empirical tests of this prediction have yielded mixed results (Glöckner and Herbold, 2011; Stewart et al., 2016).

### 4.3. Strategic determinants of attention

Strategic factors might also guide attention allocation. Heuristic strategies describe how choice processes can be simplified, often by focusing on specific attributes (Thorngate, 1980; Payne et al., 1993). For instance, the minimax heuristic (Savage, 1954) makes decisions by comparing the least attractive outcome of each option; the maximax heuristic (Thorngate, 1980) compares the most attractive outcomes. Both heuristics ignore probabilities. Their attentional policies imply different risk attitudes, with minimax leading to risk-averse and maximax to risk-seeking choices, reflected in distinct shapes of psychoeconomic functions (Pachur et al., 2017).

Other heuristics (e.g., the priority heuristic, the lexicographic heuristic; Payne et al., 1993; Brandstätter et al., 2006) predict a sequential inspection of attributes and that search is stopped as soon as the options differ on a given attribute. Heuristics might thus serve as a starting point for predicting patterns in attention allocation, and exploring how heuristic strategies are selected depending on the structure of the choice problem might illuminate how attentional policies vary across trials (Lieder and Griffiths, 2017; Mohnert et al., 2019).

## 5. Conclusion

For decades, a key approach to developing descriptive models for decision making under risk has been to modify a normative model, EV theory, by introducing transformations that distort the attributes of risky options (i.e., outcomes and probabilities) such that the predicted decisions match the observed ones. The psychological processes underlying the observed decisions were neither modeled nor measured. In this article we described an alternative approach. In process-level theories, deviations from EV maximization are not modeled by distorting outcome or probability information; instead, they are explained by a directly measurable aspect of cognitive processing: attention allocation. The development toward more cognitively grounded, attentional explanations of deviations from EV or EU maximization can be viewed as an “attentional turn” in research on risky choice. We argued that attention-based theoretical accounts of risky choice should not only predict how attention allocation shapes the accumulation of evidence, but also how patterns in attention allocation arise (for an example of how this might be achieved, see Johnson and Busemeyer, 2016).

As Herbert Simon noted, “attention is a major scarce resource” and people “cannot afford to attend to information simply because it is there” (Simon, 1978, p. 11). As researchers, “we must give an account not only of substantive rationality—the extent to which appropriate courses of action are chosen—but also procedural rationality—the effectiveness, in light of human cognitive powers and limitations, of the procedures used to choose actions” (Simon, 1978, p. 9). With the attentional turn we have outlined, research in risky choice might finally take on Simon's call and account for the intricate interplay between attention and preference.

## Data availability statement

The original contributions presented in the study are included in the article/supplementary material, further inquiries can be directed to the corresponding author.

## Author contributions

VZ and TP: conceptualization, writing, and editing. Both authors contributed to the article and approved the submitted version.

## Funding

The Max Planck Society provided funding for the Article Processing Fees for this article.

## Conflict of interest

The authors declare that the research was conducted in the absence of any commercial or financial relationships that could be construed as a potential conflict of interest.

## Publisher's note

All claims expressed in this article are solely those of the authors and do not necessarily represent those of their affiliated organizations, or those of the publisher, the editors and the reviewers. Any product that may be evaluated in this article, or claim that may be made by its manufacturer, is not guaranteed or endorsed by the publisher.
